# Supplementary material for: Pyruvate Dehydrogenase Kinase 1 inhibition mediated oxidative phosphorylation enhancement in cartilage promotes osteoarthritis progression
Source: BMC Musculoskelet Disord. 2023 Jul 20;24:597. doi: 10.1186/s12891-023-06585-6 (PMC10357736; doi:10.1186/s12891-023-06585-6)
Supplement: Supplementary file 1 — Additional file 1. [file 12891_2023_6585_MOESM1_ESM.zip › BP.Gsea.1653623667859/GOBP_BILE_ACID_BIOSYNTHETIC_PROCESS.html]

Details for gene set GOBP\_BILE\_ACID\_BIOSYNTHETIC\_PROCESS[GSEA]

|  || Dataset | 55457\_collapsed\_to\_symbols.cls.cls #OA\_versus\_normal.cls.cls #OA\_versus\_normal\_repos |
| Phenotype | cls.cls#OA\_versus\_normal\_repos |
| Upregulated in class | OA |
| GeneSet | GOBP\_BILE\_ACID\_BIOSYNTHETIC\_PROCESS |
| Enrichment Score (ES) | 0.40777904 |
| Normalized Enrichment Score (NES) | 1.4989625 |
| Nominal p-value | 0.039534885 |
| FDR q-value | 0.37682027 |
| FWER p-Value | 1.0 |
Table: GSEA Results Summary

  

Fig 1: Enrichment plot: GOBP\_BILE\_ACID\_BIOSYNTHETIC\_PROCESS      
 Profile of the Running ES Score & Positions of GeneSet Members on the Rank Ordered List

  

| SYMBOL | TITLE | RANK IN GENE LIST | RANK METRIC SCORE | RUNNING ES | CORE ENRICHMENT || 1 | SCP2 | sterol carrier protein 2 [Source:HGNC Symbol;Acc:HGNC:10606] | 105 | 0.905 | 0.1262 | Yes |
| 2 | CYP27A1 | cytochrome P450 family 27 subfamily A member 1 [Source:HGNC Symbol;Acc:HGNC:2605] | 611 | 0.619 | 0.1790 | Yes |
| 3 | AMACR | alpha-methylacyl-CoA racemase [Source:HGNC Symbol;Acc:HGNC:451] | 749 | 0.581 | 0.2547 | Yes |
| 4 | CES1 | carboxylesterase 1 [Source:HGNC Symbol;Acc:HGNC:1863] | 918 | 0.537 | 0.3215 | Yes |
| 5 | ACOT8 | acyl-CoA thioesterase 8 [Source:HGNC Symbol;Acc:HGNC:15919] | 1088 | 0.500 | 0.3825 | Yes |
| 6 | OSBPL9 | oxysterol binding protein like 9 [Source:HGNC Symbol;Acc:HGNC:16386] | 1564 | 0.418 | 0.4078 | Yes |
| 7 | SLC27A5 | solute carrier family 27 member 5 [Source:HGNC Symbol;Acc:HGNC:10999] | 3525 | 0.200 | 0.2852 | No |
| 8 | CYP7B1 | cytochrome P450 family 7 subfamily B member 1 [Source:HGNC Symbol;Acc:HGNC:2652] | 3584 | 0.195 | 0.3096 | No |
| 9 | CYP39A1 | cytochrome P450 family 39 subfamily A member 1 [Source:HGNC Symbol;Acc:HGNC:17449] | 3725 | 0.183 | 0.3260 | No |
| 10 | OSBPL2 | oxysterol binding protein like 2 [Source:HGNC Symbol;Acc:HGNC:15761] | 3906 | 0.164 | 0.3363 | No |
| 11 | HSD17B10 | hydroxysteroid 17-beta dehydrogenase 10 [Source:HGNC Symbol;Acc:HGNC:4800] | 4060 | 0.150 | 0.3467 | No |
| 12 | PROX1 | prospero homeobox 1 [Source:HGNC Symbol;Acc:HGNC:9459] | 4299 | 0.128 | 0.3473 | No |
| 13 | CYP7A1 | cytochrome P450 family 7 subfamily A member 1 [Source:HGNC Symbol;Acc:HGNC:2651] | 4499 | 0.113 | 0.3486 | No |
| 14 | CYP46A1 | cytochrome P450 family 46 subfamily A member 1 [Source:HGNC Symbol;Acc:HGNC:2641] | 4785 | 0.088 | 0.3396 | No |
| 15 | STAR | steroidogenic acute regulatory protein [Source:HGNC Symbol;Acc:HGNC:11359] | 4805 | 0.087 | 0.3510 | No |
| 16 | OSBPL3 | oxysterol binding protein like 3 [Source:HGNC Symbol;Acc:HGNC:16370] | 4912 | 0.079 | 0.3545 | No |
| 17 | SLC27A2 | solute carrier family 27 member 2 [Source:HGNC Symbol;Acc:HGNC:10996] | 5122 | 0.062 | 0.3475 | No |
| 18 | FGFR4 | fibroblast growth factor receptor 4 [Source:HGNC Symbol;Acc:HGNC:3691] | 5351 | 0.044 | 0.3363 | No |
| 19 | AKR1D1 | aldo-keto reductase family 1 member D1 [Source:HGNC Symbol;Acc:HGNC:388] | 5836 | 0.006 | 0.2996 | No |
| 20 | AKR1C4 | aldo-keto reductase family 1 member C4 [Source:HGNC Symbol;Acc:HGNC:387] | 5843 | 0.005 | 0.3000 | No |
| 21 | OSBPL1A | oxysterol binding protein like 1A [Source:HGNC Symbol;Acc:HGNC:16398] | 6615 | -0.051 | 0.2476 | No |
| 22 | NR1D1 | nuclear receptor subfamily 1 group D member 1 [Source:HGNC Symbol;Acc:HGNC:7962] | 6974 | -0.078 | 0.2314 | No |
| 23 | ABCB11 | ATP binding cassette subfamily B member 11 [Source:HGNC Symbol;Acc:HGNC:42] | 7825 | -0.143 | 0.1866 | No |
| 24 | OSBP | oxysterol binding protein [Source:HGNC Symbol;Acc:HGNC:8503] | 7884 | -0.147 | 0.2038 | No |
| 25 | NR1H4 | nuclear receptor subfamily 1 group H member 4 [Source:HGNC Symbol;Acc:HGNC:7967] | 8481 | -0.192 | 0.1861 | No |
| 26 | ABCD3 | ATP binding cassette subfamily D member 3 [Source:HGNC Symbol;Acc:HGNC:67] | 8877 | -0.223 | 0.1885 | No |
| 27 | SIRT1 | sirtuin 1 [Source:HGNC Symbol;Acc:HGNC:14929] | 8947 | -0.227 | 0.2170 | No |
| 28 | OSBPL7 | oxysterol binding protein like 7 [Source:HGNC Symbol;Acc:HGNC:16387] | 8971 | -0.230 | 0.2493 | No |
| 29 | BAAT | bile acid-CoA:amino acid N-acyltransferase [Source:HGNC Symbol;Acc:HGNC:932] | 10523 | -0.376 | 0.1847 | No |
Table: GSEA details [plain text format]

  

Fig 2: GOBP\_BILE\_ACID\_BIOSYNTHETIC\_PROCESS      
 Blue-Pink O' Gram in the Space of the Analyzed GeneSet

  

Fig 3: GOBP\_BILE\_ACID\_BIOSYNTHETIC\_PROCESS: Random ES distribution      
 Gene set null distribution of ES for **GOBP\_BILE\_ACID\_BIOSYNTHETIC\_PROCESS**
